# Photonics and Nanophotonics and Information and Communication Technologies in Modern Food Packaging

**DOI:** 10.1186/s11671-015-0939-7

**Published:** 2015-05-23

**Authors:** Olha Sarapulova, Valentyn Sherstiuk, Vitaliy Shvalagin, Aleksander Kukhta

**Affiliations:** 1 Institute of Publishing and Printing, National Technical University of Ukraine “Kyiv Polytechnic Institute”, Kyiv, Ukraine; 2 L.V. Pisarzhevsky Institute of Physical Chemistry of the National Academy of Science of Ukraine, Kyiv, Ukraine; 3 Research Institute for Nuclear Problems of Belarusian State University, Minsk, Republic of Belarus

**Keywords:** 78.67.Sc, 75.75.Cd, 82.35, ICT, Printed packaging, Smart packaging, Luminescent-magnetic nanocomposites, Zinc oxide, Safety of food

## Abstract

The analysis of the problem of conjunction of information and communication technologies (ICT) with packaging industry and food production was made. The perspective of combining the latest advances of nanotechnology, including nanophotonics, and ICT for creating modern smart packaging was shown. There were investigated luminescent films with zinc oxide nanoparticles, which change luminescence intensity as nano-ZnO interacts with decay compounds of food products, for active and intelligent packaging. High luminescent transparent films were obtained from colloidal suspension of ZnO and polyvinylpyrrolidone (PVP). The influence of molecular mass, concentration of nano-ZnO, and film thickness on luminescent properties of films was studied in order to optimize the content of the compositions. The possibility of covering the obtained films with polyvinyl alcohol was considered for eliminating water soluble properties of PVP. The luminescent properties of films with different covers were studied. The insoluble in water composition based on ZnO stabilized with colloidal silicon dioxide and PVP in polymethylmethacrylate was developed, and the luminescent properties of films were investigated. The compositions are non-toxic, safe, and suitable for applying to the inner surface of active and intelligent packaging by printing techniques, such as screen printing, flexography, inkjet, and pad printing.

## Background

Information and communication technologies (ICT) determine the current status of many areas of human activity, including industry, science, and culture. ICT is currently the nervous system of society. According to economists, about 80 % innovation and 40 % performance improvements is connected to the initiation and use of ICT [1].

Digital convergence, i.e., the combination of existing and new technologies in ICT, is a source of innovations that lead to breakthroughs in many areas of scientific, technical, and business activities. Photonics and nanophotonics have played and will play critical role in conjunction with ICT. It is worth mentioning that the term “photonics” is ambiguous: on the one hand, it is understood as a complex of photochemical and photophysical properties of molecular systems in electronic-excited state; on the other hand, it is known as both molecular and technical systems in which carriers are not electrons as in electronics, microelectronics, nanoelectronics, but photons [2]. The combination of electronics and photonics currently seems to be one of the most fruitful areas of scientific and technological progress, for example, LED technology with both semiconductor and organic and inorganic nanoscale molecular systems [3–5]. Among the latter oxides of transition metals, particularly iron and zinc sulfides and generally chalcogenide nanoparticles and metals are worthy of attention as precious and those that have unfilled electronic structure [6, 7]. Photonics of these systems allows recording information on the environment in which excited molecules and nanoparticles are placed. In particular, it opens the path of creation of information systems that can be used as sensors in modern packaging of food and agriculture products in general. Since these sensor systems can be administered in packaging material or on its surface printing methods are real development of so-called active and intelligent packing, which require advanced Agrokultura and packaging industry.

This paper is devoted to experimental investigation of photonics of nanoscale zinc oxide composites in the systems for perspective-printed active and smart food packaging. The created compositions based on nano-ZnO in the form of printing inks are suitable for manufacturing of new nanophotonic devices for packaging by printing techniques. It will be necessary to ensure that the structure of created compositions adjusts with variables of manufacturing process using printing techniques (including gravure and flexographic printing) of nanophotonic devices that will ensure the functionality of novel food packaging. The match of materials and printing process parameters will ensure printed nanophotonic elements to have sufficient photophysical characteristics, which in perspective are to be registered by sensor devices with special software; the feedback of sensors will give information of the change of photophysical characteristics of nanophotonic elements, which will reveal the processes inside the package, i.e., packaged food product state and suitability for consumption. This is the connection between ICT and the experimental part, which deals with nanophotonic layers for novel food packaging. Thus, smart packaging with nanophotonic-printed elements is to enhance product consumption culture and safety.

### Background: ICT and Photonics in Packaging Industry

ICT take priority among the most important areas of research and development projects and their implementation. Topical approach to problem solving of packaging industry uses ICT achievements. Trends in the development of modern food packaging technologies are related to active and intelligent packaging. The issue of authenticity of food products is extremely important, as well as diagnosis of flawed and damaged provisions, e.g., development of methodology of detection of the causes of food defects such as discoloration, aftertastes, and precipitates in drinks. Evaluation of shelf life and enhancing stability of food are becoming increasingly important. The role of ICT cannot be overestimated in the realization of these trends and plans, which should really be a breakthrough in the packaging industry and agricultural production and achieve food safety and general safety of life. Research is currently aimed at supporting the industry in the implementation and management of quality assurance and food safety [8].

The solution to the issues discussed may be associated in some way with the introduction of nanotechnology and ICT in the field of printing and packaging area of agricultural and food industry, particularly for the safety of packaged food and providing information about its condition at different levels—for consumers, traders, manufacturers, and logistic units. Experts in the field of nanotechnology have expressed the opinion that “nanotechnology revolution will do the same in the manipulation of matter that computers have done in manipulating information” [9]. The revolutionism of nanotechnology and its prevalence in the future is well illustrated by the words of Nobel laureate Jaures Alferov: “Nanotechnology is an absolute technology that ensures progress in all known applications from the earth to space” [10]. Since nanotechnology, according to experts, can generate risk factors and challenges, the role of ICT in the packaging industry is extremely important. It can be argued that ICT in this area should accompany any stage of technological development.

Printed packaging is considered to be one of the promising solutions for consumer packaging which can be combined with ICT. It employs printing or coating technology for introducing the components of sensory systems that indicate the status and quality of a packaged product. Among these system tags that contain substances whose color under visible or UV light correlates to the changes in quality of packaged products can be very effective. Since sensitivity of the fluorescent emission techniques significantly exceeds absorption spectroscopy ones, registration of light emission signals can effectively report the state of packaged food products, and ICT techniques can transmit this information to a particular user or monitoring system. It is promising to place data carriers for new generation packaging into packaging material or on its surface, both external and internal, by printing techniques.

### Luminescent Nanocomposites and Perspectives of ICT for Ensuring Quality Control of Food Products

The research was carried out to determine the possibility to use luminescent nanosized systems for registration of changes that take place in packaged food products during storage. We studied the influence of substances that emerge in food products as a result of spoilage processes (lactic and acetic acids, alcohols, amines) on luminescence of nanosized zinc oxide (ZnO) and organic luminophores. We created nanophotonic compositions and researched their properties in order to use them for the manufacture of printed novel packaging. Nanophotonic systems as printed elements are promising to ensure the functionality of novel packaging—active and intelligent (smart) packaging, which inform on the status of a packaged food product by changing the properties of an external or internal printed elements or sensors (optical, mechanical, electronic, or other properties). These changes are to be registered visually or instrumentally, by internal of external devices (sensors), in the latter case enabling combination with ICT, as the response of packaging can be transformed into electrical signals and processed and analyzed or passed into computer systems for processing. Therefore, creating nanophotonic compositions and future simulation and study of the characteristics of printing techniques of producing printed elements and functional surfaces, including multilayer, containing these system is an actual task. It will enable cost- and energy-efficient creation of active and intelligent packaging merged with ICT, whose mechanism of action is based on photonic phenomena observed in nanoscale components.

## Methods

Colloidal ZnO nanocrystals were obtained from zinc acetate ZnAc_2_ (Zn(CH_3_COO)_2_), sodium hydroxide (NaOH), and dry doubly distilled ethanol (C_2_H_5_OH) [11]. Zn(CH_3_COO)_2_ (0.183 g) was dissolved in 40 ml of ethanol, and 0.064 g of NaOH was dissolved in 10 ml of ethanol. Then, the solutions separately were cooled to 0 °C and mixed intensively. Then, the suspension was kept at 60 °C for 2 hours. The concentration of ZnO nanoparticles in the resulted colloid suspension was 2 · 10^−2^ mol/L. The average size of the nanoparticles is estimated from the diffractogram by the Scherer formula to be 5.5 nm and is consistent with the value 5.3 nm, calculated on the basis of analysis of the absorption spectra of the obtained colloid, the size distribution of the nanoparticles is estimated to be 4.6–6.0 nm [11]. ZnO nanocrystals in colloidal solution were stabilized with oil varnish and polyvinyl acetate dispersion. Nano-ZnO films were deposited onto polymer surfaces by ink-jet and screen printing. Magnetic fluid was obtained employing the method invented by E. E. Bibik [12]. Luminescent-magnetic nanocomposite material was synthesized from colloidal suspension of ZnO and magnetic fluid (nanosized Fe_3_O_4_). Polystyrene was used as a polymer matrix.

Amines such as dopamine, tyramine, tryptamine, and histamine were used as model substances which emerge in protein products during storage.

The luminescent spectra were recorded with a fluorescence spectrometer (LS 55; Perkin Elmer, Waltham, MA, USA). The absorption spectra (optical density) were recorded with a spectrophotometer (Specord 210; Analytik Jena, Jena, Germany).

## Results and Discussion

### Luminescence in Colloidal Solutions

**Fig. 1:**
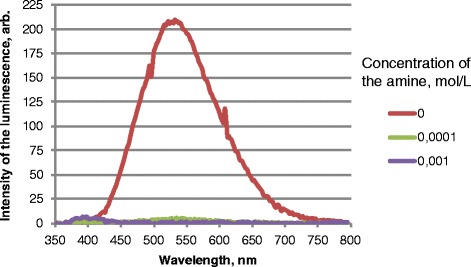
Luminescence spectrum of nano ZnO and its quenching by dopamine, *λ*_ex._ = 330 nm

It was discovered that nano-ZnO with concentration of 2 · 10^−3^ mol/L change luminescence significantly under the influence of dopamine is the most significant (Fig. 1).

**Fig. 2:**
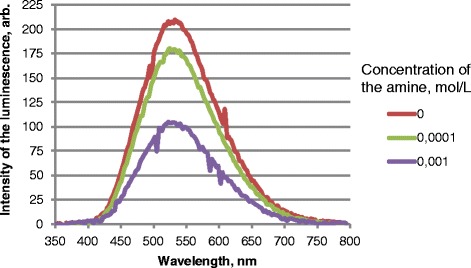
Luminescence spectra of nano ZnO without and in presence of 0.0001 and 0.001 mol/L of tryptamine, *λ*_ex._ = 330 nm

Under the influence of other metabolic amines—tyramine, tryptamine, and histamine—luminescence intensity of the samples decreased gradually (Fig. 2) [13].

**Fig. 3:**
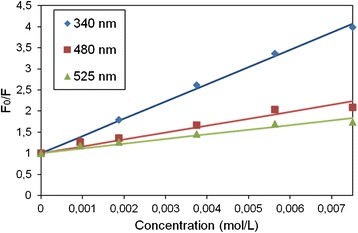
Stern-Volmer dependencies of the ratio of luminescence intensities on the concentration of quencher for different wavelengths of excitation. Luminescent-magnetic nanocomposites (nano-ZnO and magnetite in polystyrene matrix) were used for obtaining the dependencies, magnetite serving as a quencher

Luminescence quenching obeys laws [14, 15], which can be displayed as a Stern-Volmer dependency in coordinates *F*_0_/*F*—concentration of a quencher (Fig. 3, in luminescent-magnetic nanoparticles magnetite serves as a quencher), where *F*_0_ and *F* are the intensity of luminescence (with a constant *k*_1_) in the absence (unimolecular process with a constant of luminescence deactivation *k*_2_) and in the presence of a quencher (bimolecular process with a constant of quenching *k*_3_), respectively. It is known that the ratio of the quantum yield of luminescence of a luminophore and the system of a luminophore and quencher A *φ* and *φ*_A_ is as follows:

1 $$ \varphi /{\varphi}_A=1 + k2\tau \left[A\right] $$

where *φ* and *φ*_A_ are the quantum yields of pure luminophore and a system luminophore-quencher A; *τ* is the measured lifetime of an excited molecule of luminophore or a nanoparticle, which is reciprocal of the sum of monomolecular constants:

2 $$ \tau =1/\left({k}_{\mathsf{1}}+{k}_{\mathsf{3}}\right) $$

In our experiments, satisfactory straightening of experimental curves is achieved in the coordinates “relative luminescence intensity—concentration of quencher.” This allows us to objectively evaluate the sensor system data and transmit it to ICT systems.

Assuming that the ratio of the quantum yield can be replaced by the ratio of intensities of luminescence of pure luminophore and luminophore-quencher system (in this case, luminescent-magnetic nanocomposites based on nano-ZnO, magnetite and polystyrene as a matrix, optical density of the material is less than 0.1 in the visible region of the spectrum) and build the ratios *F*_0_/*F*—quencher concentration, the slope of the straight sections can be used to determine lifetime (or relative lifetime) of excited luminophore molecules (nanocomposite) in the presence of substances that affect quality of packaged food.

Thus, ICT tools can monitor the quality of packaged products, monitoring signals received as a result of irradiation of packaging or measuring absorption of light in the specified range of the electromagnetic spectrum as nanosized ZnO on printed tags also decreases intensity of absorption (in the area of 300–340 nm) in contact with amines. In this process, organic-inorganic composites with enhanced absorption are formed, although in this case, only in the shortwave spectrum area.

For samples with nanoscale organic luminophores with concentration of 10^−5^ mol/L when exposed to histamine, tyramine, and ahmatynom, there was a significant increase in luminescence intensity of the samples (2–2.75 times), whereas in the presence of dopamine and tryptamine, the increase in luminescence intensity was relatively small (1.1–1.5 times) and in the presence of norepinephrine, luminescence quenching was generally observed. Similar results were obtained for polyvinylpyrrolidone (PVP)-based films with nano-ZnO nanoparticles [13].

Thus, it investigated the emission characteristics of nanoscale inorganic (ZnO) and organic (e.g., rhodamine) luminophores in contact with amines, and it determined the correlation between the concentration of substances that indicate spoilage of a packaged food product and changes in the intensity of luminescence of created indicator systems. These components can be used to create nanophotonic and photocatalytic systems for modern printed packaging.

### Luminescence in Polymer Matrices and Printed Surfaces

It is very important to stabilize nanocomposites in solid condensed phase and apply fluorescent nano-ZnO films for their subsequent use in active and intelligent packaging systems. Considering that optimization of luminescent compositions and conditions for the application of the compositions to surfaces was carried out. There was found a distinctive influence of several factors on the luminescent characteristics and the intensity of luminescence of films that are made of prepared colloidal solutions containing zinc oxide nanocomposites.

Introduction of ZnO nanoparticles to a polymer for application of the composition to packaging material usually lead to the loss of luminescent properties of polymer films. The choice of macromolecular compounds is limited by the fact that high luminescent stable ZnO nanocrystals has been obtained in a limited range of solvents such as ethanol, isopropanol, and dimethylformamide [11]. Besides, the polymer has to be non-toxic and do not interact with food product to be packaged.

The following polymers were investigated: polymethylmethacrylate (PMMA), polyvinyl alcohol (PVA), gelatin, polyvinylpyrrolidone (PVP). It was revealed that the introduction of nano-ZnO to PMMA, PVA, and gelatin leads to the loss of luminescent properties, and only with the use of PVP can stable fluorescent compositions retain fluorescent properties after application to the surface and evaporation of the solvent. It is important that PVP is safe and is used in the food industry as a food additive E1201, as well as in medical practice PVP solutions are known as temporary blood substitutes. Another advantage of PVP is the fact that PVP allows obtaining transparent coatings. Therefore, in this paper, we investigated the formation of films of ZnO nanoparticles in PVP and the influence of several factors on the intensity of luminescence of these films.

PVP was added to the ZnO colloidal solution in ethanol (concentrations 2 · 10^−3^, 1 · 10^−2^, and 2 · 10^−2^ mol/L) at room temperature and stirring (molar mass 10,000, 40,000, and 360,000 g/mol) to obtain PVP concentration of 10 and 25 % in solution, then the composition was deposited onto glass surface (with different film thicknesses) and was left to dry at room temperature.

**Fig. 4:**
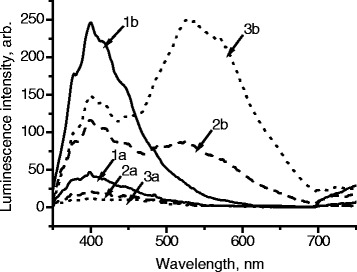
Influence of molar mass of PVP on the intensity of luminescence of films. *1 M*(PVP) = 10,000 g/mol, *2 M*(PVP) = 40,000 g/mol, *3 M*(PVP) = 360,000 g/mol, **a** Samples without ZnO. **b** [ZnO] = 2 · 10^−2^ mol/L, *λ*_ex._ = 330 nm

The influence of molar mass of PVP on stability of luminescence was investigated. It was found that with the increase of molar mass of PVP, luminescence intensity of samples increases (Fig. 4).

The described changes can be explained by the participation of functional groups at the ends of the polymer chains of PVP in radiative processes in the polymer, as well as effective capture of electronic excitation of ZnO nanoparticles by ending groups, followed by radiation in the natural radiation region of the polymer. With the decrease of molar mass of the polymer, the amount of such functional groups increases, which leads to the reduction of the luminescence band of nano-ZnO and the increase of luminescence intensity of PVP.

Consequently, to obtain maximum intensity of luminescence of films in the long wavelength region (which is characteristic of nanocrystalline ZnO), it is optimal to use PVP with the highest molar mass (*M* = 360,000 g/mol). As can be seen from Fig. 4, changing the molecular weight of PVP allows varying color of luminescent emission of the films from blue to green and yellow, which may be helpful for creating systems for active and intelligent packaging.

**Fig. 5:**
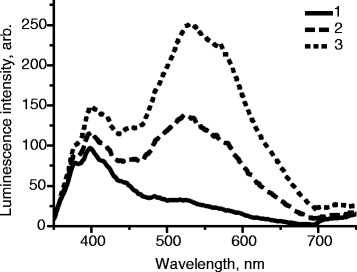
Influence of the initial concentration of colloidal ZnO on the luminescence intensity of the films. *1* [ZnO] = 2 · 10^−3^ mol/L, *2* [ZnO] = 1 · 10^−2^ mol/L, *3* [ZnO] = 2 · 10^−2^ mol/L, *λ*_ex._ = 330 nm, *M*(PVP) = 360,000 g/mol

It has been found that the luminescence intensity of PVP films with ZnO nanoparticles increases proportionally with the increase of ZnO nanoparticle concentration (Fig. 5). PVP concentration in the original solution was 25 %

It has been found that with the increase of concentration of ZnO nanoparticles luminescence intensity of PVP films increases proportionally with the increase of nanoparticle concentration (Fig. 5). PVP concentration in the original solution was 25 %.

Therefore, it is optimal to use the highest initial concentration of ZnO colloid in ethanol, which is limited to 2 · 10^−2^ mol/L [11].

**Fig. 6:**
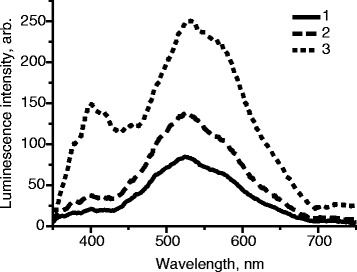
Influence of the thickness (number of layers, layer thickness is 100 μm) of PVP films containing nanosized ZnO on the intensity of luminescence, [ZnO] = 2 · 10^-2^ mol/L. *1* One layer, *2* two layers, *3* five layers, *λ*_ex._ = 330 nm, *M*(PVP) = 360,000 g/mol

The effect of film thickness of PVP containing nano-ZnO on the intensity of luminescence of films was studied, which is important for the usage of printing techniques for application of films to surfaces. Figure 6 shows the luminescence spectra of nano-ZnO films with different number of layers (simulation of different film thicknesses)—from one to five layers, layer thickness is 100 μm.

As can be seen from Fig. 6, in order to increase the intensity of luminescence of the films, film thickness of the coating layer should be increased. This is possible through the use of printing techniques—screen printing, flexography, inkjet, pad printing, etc.

**Fig. 7:**
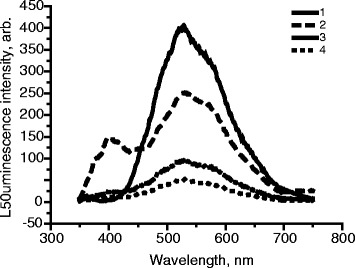
*1* Luminescence of ZnO nanoparticles, *2* uncoated films containing ZnO nanoparticles PVP, and *3* films coated with 1 % PVA solution and *4* 10 % PVA solution, *λ*_ex._ = 330 nm, layer thickness is 10 μm

A disadvantage of PVP is its solubility in water, which makes it difficult to use these films in contact with foods which contain water. To overcome this shortcoming, the authors suggest the use of a thin coating layer of PVA (layer thickness is up to 10 μm). It has been found that the use of such coatings as polyurethane or butyl acetate is not possible since they result in almost complete quenching of luminescence. PVA is a water soluble polymer, however, after drying it becomes insoluble in water due to the formation a network structure. The effect of the coating of PVA on the intensity of the luminescence of zinc oxide films in PVP was studied. For varying the thickness of the coating, PVA solutions with different concentrations were applied to the surface of the ZnO in PVP using “dip-coating” technique. It is possible to adjust the thickness of the coatings more conveniently using printing techniques. According to Fig. 7 (curves 3 and 4) PVA coating leads to significant luminescence quenching, but if the coating thickness is reduced, luminescence quenching of the films can be reduced too. Thus, it is reasonable to use thin layers of PVA (with layer thickness up to 10 μm) coating of the films if they are to be placed in contact with water.

Due to the fact that the use of PVA coatings for imparting fluorescent films of water resistance leads to deterioration of the luminescent properties of the films, options of stabilization of ZnO nanoparticles for subsequent introduction of them to the water-resistant polymers were considered. In particular, the results were obtained which indicate the prospects of stabilizing ZnO nanoparticles with silicic acid anhydride and PVP with following introducing them to PMMA. For this purpose, colloidal ZnO nanocrystals in ethanol were introduced to PVP and silicic acid anhydride, and the resulting solution was mixed with PMMA solution in 1,2-dichloroethane with vigorous stirring at room temperature. 1,2-Dichloroethane was used as a solvent for PMMA, the solvent is vaporized after the application of the composition to packaging material and in future needs to be replaced because of the subsequent use of the composition for food packaging. The prerequisite of preserving luminescent properties of ZnO nanoparticles in PMMA in our study was the simultaneous presence of silicic acid anhydride and PVP as stabilizers of nano-ZnO. The absence of silicic acid anhydride or PVP in the system resulted in complete suppression of luminescence of ZnO nanoparticles after drying PMMA film. This synergistic effect can be explained by better stabilization of the surface defects of ZnO nanocrystals, adsorbed in the pores of silicic acid anhydride.

**Fig. 8:**
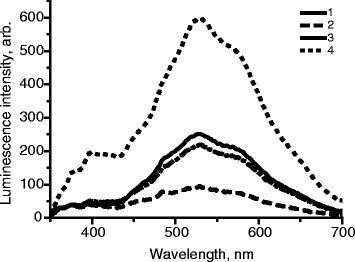
Effect of the concentration of ZnO nanoparticles, stabilized by silicic acid anhydride and PVP in PMMA on the luminescence intensity of films obtained by drying these compositions on glass substrate. *1* film of nano-ZnO, stabilized by silicic acid anhydride and PVP without PMMA, [ZnO] = 2 · 10^−2^ mol/L, *2* [ZnO] = 6,67 · 10^−3^ mol/L, *3* [ZnO] = 1 · 10^−2^ mol/L, *4* [ZnO] = 1,33 · 10^−2^ mol/L, *λ*_ex._ = 330 nm

Compositions in which the concentration of nano-ZnO in an alcohol solution containing silicic acid anhydride, PVP, PMMA, and dichloroethane varied were deposited onto glass substrates to obtain the films. Their luminescence spectra are represented in Fig. 8.

As can be seen from Fig. 8, as concentration of ZnO in the starting mixture is increased from 6.67 · 10^−3^ to 1.33 · 10^−2^ mol/L, the intensity of luminescence in long wavelength region of spectrum of ZnO nanoparticles with a maximum of 520 nm increases from 100 to 600 AU. Moreover, the last value is more than twice the intensity of the luminescence as nano-ZnO stock solution in ethanol or in PVP films. The latter can be explained by the fact that in colloidal solution or PVP film ZnO nanoparticles may be partially aggregated. As in the investigated particles, quantum size effects take place, namely the dependence of the band gap of the semiconductor on the nanoparticles size; and in such systems, there is always a size distribution, charge separation between nanoparticles of different sizes is possible that ultimately leads to reduction in the intensity of luminescence. In the case of the additional use of silicic acid anhydride in the system, ZnO particles are distributed over its surface and pores, resulting in no direct contact between them and consequential increase of luminescence intensity.

## Conclusions

As a result of the experimental study, luminescent films based on nanosized zinc oxide were obtained for the usage in active and intelligent (smart) packaging. On the basis of study of the influence of molar mass and concentration of PVP, the concentration of ZnO nanoparticles and layer thickness of the films, optimal composition, and parameters of application of luminescent composition to surfaces was developed.

The possibility of coating of luminescent films based on ZnO nanoparticles and water-soluble PVP to eliminate solubility of the obtained films was investigated. It was found that thin layer of 1 % PVA solution in water has the least significant effect on the intensity of luminescence of the nano-ZnO films. The composition has been developed for obtaining insoluble in water highly luminescent films based on ZnO nanoparticles, stabilized by silicic acid anhydride and PVP in PMMA. The optimal composition of the components was developed and its luminescent properties were studied.

Thus, the luminescent compositions based on zinc oxide nanoparticles were obtained for application to packaging materials to create active and intelligent (smart) packaging which is able to inform the consumer of status of a packaged food product by varying the intensity of luminescence. For applying the designed compositions to packaging material such as polypropylene film, it is promising to use printing techniques such as flexography, screen, inkjet, and pad printing.

Before the film can be used in food packaging, further research is needed. It will be necessary to ensure that structure of created compositions adjusts with variables of manufacturing process using printing techniques of nanophotonic devices that will ensure the functionality of novel food packaging. It will be necessary to study the application process of the developed printing inks with nanophotonic components to paper and polymer substrates, to optimize existing and develop new efficient printing techniques of formation of functional elements on the surface and inside multilayer materials of active and intelligent packaging and to solve the problem of adjustment of materials variables (substrate parameters and nanophotonic composition contents and viscosity) and printing process variables of printed nanophotonic elements for active and intelligent (smart) food packaging.

The analysis of the problem of conjunction of information and communication technologies with packaging industry and food production shows the importance of setting general and specific objectives of the packaging industry to researchers and developers of new packaging technology, food production workers, distributors, and consumers of packaged products. Combining the latest advances in the development of packaging industry, nanotechnology, including nanophotonics and ICT, stimulates new developments and progress in the packaging business and related fields.

## References

[1] Ezell S, Scott A (2010). ICT R&D policies: an international perspective. Internet Computing, IEEE.

[2] Saleh B, Malvin CT, Bahaa ES (1991). Fundamentals of photonics.

[3] Sherstyuk VP, Sarapulova OO, Shvalagin VV. Luminescent hybrid nanocomposites and prospects of molecular and nanophotonic systems in modern packaging and printing. Repino, St. Peterburg, Russia: Book of Abstracts of the 3-International Symposium “Molecular Photonics”; 2012. p. 79.

[4] Yariv A, Pochi Y (2006). Photonics: optical electronics in modern communications (the oxford series in electrical and computer engineering).

[5] Ruoxue Y, Gargas D, Yang P (2009). Nanowire photonics. Nat Photonics.

[6] Biswas K, Rao C (2007). Use of ionic liquids in the synthesis of nanocrystals and nanorods of semiconducting metal chalcogenides. Chem A Eur J.

[7] Rao CN (2005). Use of the liquid–liquid interface for generating ultrathin nanocrystalline films of metals, chalcogenides, and oxides. J Colloid Interface Sci.

[8] Gelb E, Parker C. Is ICT adoption for agriculture still an important issue. http://departments.agri.huji.ac.il/economics/gelb-gelb-parker.pdf. Accessed 5 September 2014.

[9] Mansoori GA, Fauzi TA (2005). Nanotechnology-an introduction for the standards community. J ASTM Int.

[10] Vasiutina V, Sherstiuk VP (2010). Problems of printing and packaging industry in the light of the achievements of nanotechnology. Technol Tech Printing.

[11] Shvalagin VV, Stroyuk AL, Kuchmii SY (2004). Role of quantum-sized effects on the cathodic photocorrosion of ZnO nanoparticles in ethanol. Theor Exp Chem.

[12] Bibik EE, Bibik EE (1973). Preparation of a ferrofluid. Kolloidnyi Zhurnal. 1973;35:1141–2. transl. in. Colloid Journal of the USSR.

[13] Sarapulova O Kyrychok T, Sherstiuk V, Orlov A. Modern printing technologies for micro- and nanoelectronics. Proceedings of IEEE XXXIII International Scientific Conference Electronics and Nanotechnology (ELNANO) April 16–19, 2013 Kyiv, Ukraine 2013: 151–155.

[14] Turro N. Molecular photochemistry. Moscow: Mir; 1967. in Rus.

[15] Calvert J, Pitts J. Photochemistry. Moscow: Mir; 1968. in Rus.

