# Erratum to: Photonics and Nanophotonics and Information and Communication Technologies in Modern Food Packaging

**DOI:** 10.1186/s11671-017-1942-y

**Published:** 2017-03-09

**Authors:** Olha Sarapulova, Valentyn Sherstiuk, Vitaliy Shvalagin, Aleksander Kukhta

**Affiliations:** 1 Institute of Publishing and Printing, National Technical University of Ukraine “Kyiv Polytechnic Institute”, Kyiv, Ukraine; 2 L.V. Pisarzhevsky Institute of Physical Chemistry of the National Academy of Science of Ukraine, Kyiv, Ukraine; 3 Research Institute for Nuclear Problems of Belarusian State University, Minsk, Republic of Belarus

## Erratum

In the original publication [1] two Google Scholar links were missing in the reference list. The complete references can be found here:

3. Sherstyuk VP, Sarapulova OO, Shvalagin VV. Luminescent hybrid nanocomposites and prospects of molecular and nanophotonic systems in modern packaging and printing. Repino, St. Peterburg, Russia: Book of Abstracts of the 3-International Symposium “Molecular Photonics”; 2012. p. 79.

https://scholar.google.com.ua/scholar?hl=uk&q=Luminescent+hybrid+nanocomposites+and+prospects+of+molecular+and+nanophotonic+systems+in+modern+packaging+and+printing&btnG=

http://onlinereg.ru/mph2012/Molecular_photonics2012_Abstracts.pdf#page=79

13. Sarapulova O Kyrychok T, Sherstiuk V, Orlov A. Modern printing technologies for micro- and nanoelectronics. Proceedings of IEEE XXXIII International Scientific Conference Electronics and Nanotechnology (ELNANO) April 16–19, 2013 Kyiv, Ukraine 2013: 151–155.

https://scholar.google.com.ua/scholar?q=Sarapulova+O+Kyrychok+T%2C+Sherstiuk+V%2C+Orlov+A.+Modern+printing+technologies+for+micro-+and+nanoelectronics.&btnG=&hl=uk&as_sdt=0%2C5
